# Root Exudates: Mechanistic Insight of Plant Growth Promoting Rhizobacteria for Sustainable Crop Production

**DOI:** 10.3389/fmicb.2022.916488

**Published:** 2022-07-14

**Authors:** Sudhir K. Upadhyay, Abhishek K. Srivastava, Vishnu D. Rajput, Prabhat K. Chauhan, Ali Asger Bhojiya, Devendra Jain, Gyaneshwer Chaubey, Padmanabh Dwivedi, Bechan Sharma, Tatiana Minkina

**Affiliations:** ^1^Department of Environmental Science, V.B.S. Purvanchal University, Jaunpur, India; ^2^Department of Biotechnology, M.H.P.G. College, Jaunpur, India; ^3^Academy of Biology and Biotechnology, Southern Federal University, Rostov-on-Don, Russia; ^4^Department of Agriculture and Veterinary Sciences, Mewar University, Chittorgarh, India; ^5^Department of Molecular Biology and Biotechnology, Maharana Pratap University of Agriculture and Technology, Udaipur, India; ^6^Cytogenetics Laboratory, Department of Zoology, Banaras Hindu University, Varanasi, India; ^7^Department of Plant Physiology, Institute of Agricultural Sciences, Banaras Hindu University, Varanasi, India; ^8^Department of Biochemistry, Faculty of Science, University of Allahabad, Allahabad, India

**Keywords:** chemoattractant, PGPR recruitment, plant-microbes interaction, root-exudate, rhizosphere

## Abstract

The breaking silence between the plant roots and microorganisms in the rhizosphere affects plant growth and physiology by impacting biochemical, molecular, nutritional, and edaphic factors. The components of the root exudates are associated with the microbial population, notably, plant growth-promoting rhizobacteria (PGPR). The information accessible to date demonstrates that PGPR is specific to the plant's roots. However, inadequate information is accessible for developing bio-inoculation/bio-fertilizers for the crop in concern, with satisfactory results at the field level. There is a need to explore the perfect candidate PGPR to meet the need for plant growth and yield. The functions of PGPR and their chemotaxis mobility toward the plant root are triggered by the cluster of genes induced by the components of root exudates. Some reports have indicated the benefit of root exudates in plant growth and productivity, yet a methodical examination of rhizosecretion and its consequences in phytoremediation have not been made. In the light of the afore-mentioned facts, in the present review, the mechanistic insight and recent updates on the specific PGPR recruitment to improve crop production at the field level are methodically addressed.

## Introduction

According to the World Health Organization (WHO), the food shortage for sustaining the human population is on a steep upward trajectory, mainly owing to the quickly booming human population that is expected to cross the 10 billion mark by 2050 (DESA UN, 2015). Both WHO and the United Nations have proposed to intensify global food production by 50% in the near future. The agriculturally important microorganisms (AIMs) can play a pivotal role in realizing this colossal target considering the fact that fertile lands are sharply shrinking owing to urbanization and industrialization. AIMs not only improve plant growth and yield but provide sustained protection against a variety of phytopathogens (Bhattacharyya and Jha, 2012; Glick, 2012; Compant et al., 2019). The beneficial microbes of the rhizosphere zone interact positively with mutually guided components of root exudates, i.e., rhizodeposits (Hassan et al., 2019). During the rhizodeposition process, the plant roots secrete carbohydrates, fatty acids, essential amino acids, organic acids, hydrolytic enzymes, growth-regulating hormones, vitamins, nucleotides, flavonoids, polyphenols, sterols, and volatile organic compounds (Hartmann et al., 2009; Hu et al., 2018; Ankati and Podile, 2019).

In the last century, the word “rhizosphere” was introduced as a microbial hot spot in the area of the rootsystem (Hartmann et al., 2008). The rhizospheric region, a specific zone around the root and harbors various kinds of microorganisms, primarily bacteria, fungi, nematodes, insect larvae, mites, amoebas, and protozoa (Bonkowski et al., 2009). The bacterial colonies residing in the rhizospheric zone are called rhizobacteria (Hartmann et al., 2009). The rhizospheric zone supports the plant root system (Ahemad and Kibret, 2014) and modulates the physico-chemical and biological properties of the soil (Ahemad and Kibret, 2014; Zhalnina et al., 2018).

The rhizosphere zone provides a shelter for the exchange of biochemical components that establish inter-species relationships between the roots and microorganisms (Gupta et al., 2020). Plant roots release various types of enzymes/compounds in the soil that mediate the interaction between microorganisms and plants (Ankati and Podile, 2019). Factors influencing soil microbial population include soil quality, soil moisture, soil pH, and rhizospheric secretion (Bagyalakshmi et al., 2012; Upadhyay and Singh, 2015; Hu et al., 2018). There are various physical and chemical parameters of the rhizospheres that impact the function of microorganisms, which ultimately affect several mechanisms, such as the respiratory process, the secretion of organic acids by the roots, the breakdown of soil organic matter, nutrient uptake, symbiotic nitrogen fixation, etc. (Reinhold-Hurek et al., 2015; Mahmud et al., 2021).

The rhizosphere plays an important role in root excretion, microbial activity, genetic exchange, improving nutrient use efficiency, and gradient diffusion, which are jointly referred to as the rhizosphere effect (Badri and Vivanco, 2009; Ladygina and Hedlund, 2010; Mendes et al., 2013). Rhizobacteria associated with the plant root are often referred to as plant growth-promoting rhizobacteria (PGPR). The functions of plant growth-promoting rhizobacteria, such as direct and indirect mechanism, metabolism, chemotaxis, secretion, antibiotic production, etc., are mediated by its gene cluster that triggers host–PGPR interactions (Mark et al., 2005; Matilla et al., 2007; Ramachandran et al., 2011; Zhang et al., 2015; Bashir et al., 2021; South et al., 2021). Ultrastructure of the root cell wall mediated PGPR interaction, which was induced by the gene expression of the plant. Ryu et al. (2003) demonstrated that out of 38 genes, 30 genes of *Bacillus subtilis*-GB03 were associated with a change in the *Arabidopsis* root-ultrastructure and promote plant growth. *Azospirillum irakense* vitalized polygalacturonase gene (PG genes) in the roots of rice plant (Sekar et al., 2000). Among PG genes, PbrPG6 is responsible for fruit-soothe (Zhang et al., 2019). The root exudation and root exudates are relevant for the survivability of plants against various environmental conditions. The root exudates aid in the selection of microbial populations around the rhizosphere (Mendes et al., 2013; Zhang et al., 2015). In the purview to tackle this aspect, the review discusses the mechanisms of root exudation, the current updates on the selective plant growth-promoting rhizobacteria aggregation and their role in plant–microbe interface, and most importantly, the future developments in plant–PGPR interactions for sustainable agriculture.

## Root Exudates and Plant Growth-Promoting Rhizobacteria

**Figure 1:**
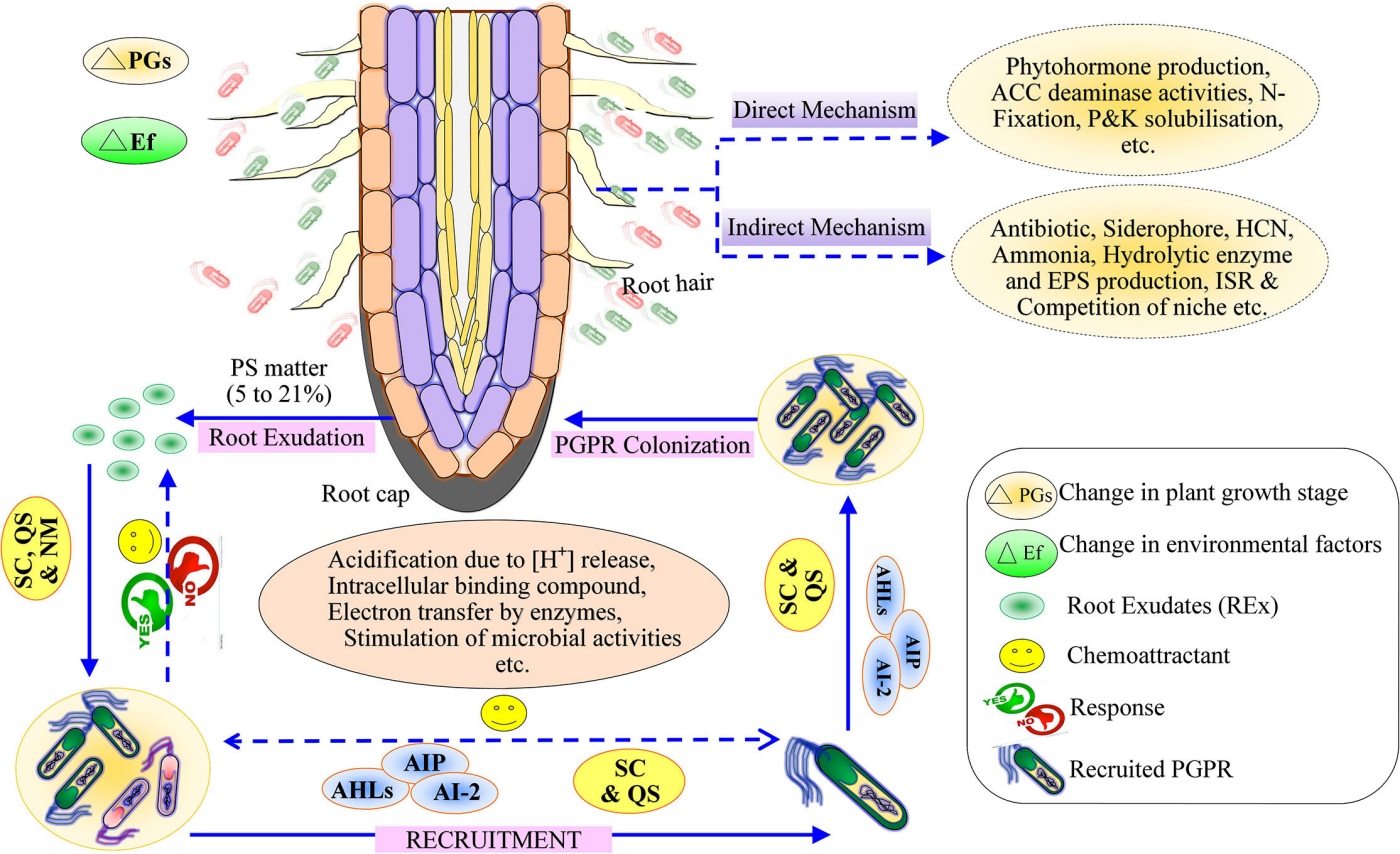
Schematic representation of the mechanism of root exudates for recruitment of plant growth-promoting rhizobacteria and plant growth-promoting mechanism (Direct and Indirect). SC, Selected compounds; QS, quorum sensing; NM, Nutrient management; AHL, Acyl Homoserine lactone; AIP, Autoinducing peptides; AI-2, Autoinducer; PS, Photosynthetic matter.

Plant root secretes 5–21% of photosynthetic matter such as carbohydrates, proteins, secondary metabolites, etc., into the rhizospheric soil environment, generally known as root exudates (Badri et al., 2013; Figure 1). The coping mechanism of plants under diverse environmental conditions mainly rests on the root acquisition of soil resources and their surroundings (Gupta et al., 2020). In the mid twentieth century, the world population increased quickly and posed various problems related to food, fiber, fuel, homeland, etc., which has consequences for hunger, poverty, water scarcity, and environmental degradation. The scarcity of food is a burgeoning challenge for humans that has been classified as goal number two of the Zero Hunger of the United Nations Sustainable Development Goals 2030.

Recently, the researchers have introduced an eco-friendly concept based on free-living bacteria called PGPR (plant-growth promoting rhizobacteria). The plant growth-promoting rhizobacteria are soil-borne or root-colonizing rhizobacteria (Upadhyay et al., 2009, 2012a,b; Singh et al., 2017; Numana et al., 2018; Upadhyay and Chauhan, 2022), which play a functional role in plant growth through several mechanisms in terrestrial ecosystems. Plant-growth promoting rhizobacteria significantly reduce the dependence on chemical fertilizers and pesticides (Liu et al., 2017). Plant growth-promoting rhizobacteria promote plant growth through root-hair proliferation, enhancing root hair branching; increase in seedling emergence; early nodulation; nodule functioning; enhanced leaf surface area; improvement in vigor and biomass; increased indigenous plant hormones levels; and most importantly, by improving nutrient use efficiency (Vocciante et al., 2022). The plant growth-promoting rhizobacteria induce the accumulation of carbohydrates in plants and consequently the yield of various plant species (Bhattacharyya and Jha, 2012; Table 1). The most dominant endophytic plant growth-promoting rhizobacteria phyla are Proteobacteria and Actinobacteria, followed by Bacteroidetes and Firmicutes (Ray et al., 2017). Endophytic bacteria enter the plant tissues by the lateral root cracks, wounds, lenticels, germinating radicles, and other parts of the plant body (Chaturvedi et al., 2016). Endophytic-rhizospheric bacteria are involved in several functions such as internal protection of the environment (Santos et al., 2018), metabolism of carbon compounds, nitrogen fixation by nitrogenase (Santoyo et al., 2016), and capability for germination of nodes (Yousaf et al., 2017).

**Table 1:** Compounds from plant root exudates recruit perfect plant growth-promoting rhizobacteria (PGPR) improving plant growth performance.

**Plant**	**Compounds from root exudates**	**Recruited PGPR**	**Plant growth performance**	**Condition**	**References**
Groundnut (*Arachis hypogaea*)	Naringenin, oleic, citric, and lactic acid	*Bradyrhizobium-Azospirillum brasilense*	Enhance root exudation and PGPR interaction	Water deficit condition	Cesari et al., 2019
	Threonine and glycoxylicoxime acid	*Pseudomonas aeruginosa* (RP2)	Enhance growth and yields of groundnut	field study	Ankati and Podile, 2019
	Serine, pentanoic acid, glycopyranoside, tartaric acid, and 2-pyrrolidinone	*Bacillus sonorensis* (RS4) and *Pseudomonas aeruginosa* (RP2)			
	Polyphenol oxidase and phenylalanine	*Pseudomonas aeruginosa* (P4)	Significantly enhance seed germination, seedling, and shoot- root length and dry weight	*in vitro*	Gupta et al., 2020
	*N*-acylhomoserine lactones (AHLs)	*Bradyrhizobia*	Induces nitrogen fixation and PGPR colonization	*in vitro*	Nievas et al., 2012
Wheat (*Triticum* sp.)	2,4 diacetylphloroglucinol (DAPG)	*Pseudomanas* (F113) *and Azospirillum* sp.	Enhances phyto-stimulation effect by *Azospirillum* Sp245-Rif (Root-proficient, spontaneous rifampicin-resistant mutant of Sp245) gene, and PGPR colonization	*in vitro*	Combes-Meynet et al., 2011
	2,4-diacetylphloroglucinol (DAPG)	*Fluorescent Pseudomonas* sp	Act as a bio-control	*in vitro*	Bonsall et al., 1997
	Organic acids (acetic acid, oxalic acid, succinic acid, and tartaric acid)	*Arthrobacter, Bacillus* and *Devosia*	Enhance the Organic compounds concentration mediates root exudation and PGPR colonization	field study	Chen et al., 2019
Rice (*Oryza sativa*)	Carbohydrates, histidine, proline, valine, alanine, and glycine	*Azospirillum brasilense*	Rice exudates significantly induce attraction of the endophytic bacteria *Corynebacterium flovesence* and *Bacillus pumilus*. *Bacillus* sp. was less attracted than endophytes while the *Azospirillum brasilense* showed higher chemotactic response	Hydrponic condition	Bacilio-Jimenez et al., 2003
	Salicylic acid (SA)	*Pseudomonas chlororaphis* (ZSB15-M2)	Increases rhizospheric colonization on foliar spray of SA or *Corynebacterium glutamicum* cell extract (CGCE) • Soil organic carbon, microbial biomass carbon, soil protein was increased with 21.86, 9.68, and 11.57%, respectively • Available form of nitrogen, phosphorus, potassium, and zinc was increase with 21.83, 28.83, 23.95, and 61.94% over the control in the rhizosphere	field study	Bowya and Balachandar, 2020
	Flavonoids and hydroxycinnamic	*Azospirillum*	Enhance metabolites activities and plant growth	field study	Chamam et al., 2013
	*N*-acyl homoserinelactones (AHLs)	*Azospirillum lipoferum* (TVV3)	Ability to enhance chemotactic interaction	*in vitro*	Vial et al., 2006
Tomato (*Solanum lycopersicum*)	Organic acids (Citric, succinic, and malic acids)	*Pseudomonas fluorescens* (WCS365)	Act as bio-control agent and increase the biomass	*In vivo*	Kamilova et al., 2006b
	Azelaic acid	*Bacillus* spp.	Acts as a bio-control through ISR and enhance plant growth performance	*in vitro*	Korenblum et al., 2020
Maize (Zea *mays*)	Humic acid	*Herbaspirillum seropedicae*	Enhances the production of border cells (involve at prime stage of plant soil ecosystem, including signaling and sense response) followed by root colonization of nitrogen fixer *Herbaspirillum seropedicae*	*in vitro*	Canellas and Olivares, 2017
	Amino acids, proline, total soluble sugar, and exopolysaccharides	*Bacillus* spp.	Enhance seedling and plant growth	Drought stress	Vardharajula et al., 2011
Pigeon pea (*Cajanus* *cajan*)	Tryptophan	*Rhizobium* spp.	IAA production that significantly enhance plant growth	*in vitro*	Ghosh et al., 2013
*Banana* (*Musa* sp.)	Oxalic, malic, furmaric, and several organic acids	*Bacillus amyloliquefaciens* (NJN-6)	Chemotactic response by malic acids and fumaric acids induced 20.7–27.3% biofilm formation	*in vitro*	Yuan et al., 2015
Common glasswort (*Salicornia europaea*)	*N*-acylhomoserine lactones (AHLs)	*Pseudomonas* *segetis* (P6)	Act as quorum quenching and bio-control agent. Increases height and weight of tomato plant	*In vivo*	Rodriguez et al., 2020
Alfalfa (*Medicago sativa* L.)	Flavonoids	*Rhizobium melilofi*	Chemoattractants and *nod* gene inducers for the symbiotic *Rhizobium*	Aeroponic system	Coronado et al., 1995
	7,4- Dihydroxyflavone and Naringeni	Acidobacteria	Induced colonization of PGPR with addition of enhancing *nod* gene expression	*in vitro*	Szoboszlay et al., 2016
	*N*-acyl homoserine lactones (AHLs)	*Sinorhizobium meliloti*	Induce nitrogen fixation and PGPR colonization	*in vitro*	Marketon et al., 2002
Soybean (*Glycine max*)	Isoflavonoid	Rhizobia	Helps in plant defense and also facilitate symbiotic interaction among soybean root and rhizobial communities	*in vitro*	White et al., 2017
Cucumber (*Cucumis sativus*)	Citric and fumaric acids	*Bacillus amyloliquefaciens* (SQR-9)	Induces colonization of *Bacillus amyloliquefaciens* SQR-9 and mitigate against pathogen *Fusarium oxysporum*	*in vitro*	Liu et al., 2014
Arabidopsis (*Arabidopsis thaliana*)	L-malic acid	*Bacillus subtilis* (FB17)	L-malic acid enhance boifilm formation chemotactically	*in vitro*	Rudrappa et al., 2008
Sugar beet (*Beta vulgaris*)	2,4-diacetylphloroglucinol (DAPG)	*Pseudomonas* spp. (F113)	Acts as a inhibitor of plant pathogens	*in vitro*	Shanahan et al., 1992

The PGPR leads to increased soil fertility, plant growth promotion, and suppression of phytopathogens. These are involved in different functions of the soil ecosystem, nutrient availability, bioremediation of toxic heavy metals, degradation of pesticides, etc. (Chandler et al., 2008; Braud et al., 2009; Rajkumar et al., 2010; Paul et al., 2020; Bhojiya et al., 2021). The PGPR induces plant growth under varied environmental conditions, and the functional roles of bacteria vary with a specific plant (Table 1). The studies demonstrated that root exudates recruited microbial species that are more favorable for plant growth and productivity (Chowdhury et al., 2015; Zhang et al., 2015).

## Plant–Microbe Interactions

Several researchers have reported that the population of microorganisms differs in the soil, for example, 12 × 10^8^ bacteria/g dry soil, 12 × 10^5^ fungi/g dry soil, 5 × 10^5^ algae/g dry soil, and 46 × 10^6^ actinomycetes/g dry soil; the bacterial population is highest followed by fungi, algae, and actinomycetes, as a general rule of the thumb (Yadav et al., 2015). Biotic interactions between the plants and microbes occur through communication that requires two essential conditions: one is the production of a specific signal, and another is the behavioral response generated from the signals (Keller and Surette, 2006). Plants communicate to rhizobacteria by secreting specific signaling molecules, viz. lectine enzymes, which are retraced by the bacterial species (Keller and Surette, 2006). *B. subtilis* detects only secondary metabolites as signals and produces a response against the secondary metabolites (Shank and Kolter, 2011; Singh et al., 2019). The behavior between the plants and the PGPR is mediated through root exudates, quorum sensing, cross-talk, electron-transfer mechanism, etc. (Tashiro et al., 2013; Singh et al., 2017; Keswani et al., 2020a). Positive plant–microbe interactions can be observed with PGPR, nitrogen-fixing bacteria, endo- and ecto-mycorrhizal fungi, whereas negative plant–microbe interactions are exhibited by pathogenic microbes (Haldar and Sengupta, 2015; Compant et al., 2019; Bashir et al., 2021; South et al., 2021). The legume rhizobia is an example of symbiotic interactions (Cai et al., 2009); the plant's secondary metabolite secretes flavonoids that activate a cascade of transcriptional events and mediates rhizobial nodulation signals commonly known as *Nod-*factors or lipo-chitooligosaccharides (Spaink, 2000). These factors trigger plant growth, leading to morphological changes in root hairs of legumes and the development of root nodules, while *Nod*-factors play a significant role in symbiotic nitrogen fixation (D'Haeze et al., 1998). The rhizospheric microbes act as biological control agents (BCA) that regulate plant pathogens. Thus, BCA ultimately increases plant productivity through the production of antimicrobial secondary metabolites (Weller, 2007; Singh et al., 2019), production of hydrolytic enzymes (Adesina et al., 2007), effectors (Rezzonico et al., 2005), and hyperparasitism (Harman et al., 2004). Plant growth-promoting microorganisms (PGPM) affect plant growth directly or indirectly through biofertilizers (Mahmud et al., 2021) and/or phytostimulators (Spaepen et al., 2007), as well as biocontrol activity (Figure 1).

The functional genes of *B. amyloliquefaciens* strains CAUB946, YAUB9601-Y2, and FZB42 are involved in the synthesis of phytohormones, and other gene clusters are involved in disease control (Chen et al., 2007; Borriss, 2011; Blom et al., 2012; Hao et al., 2012). The selection of the perfect candidate PGPR can be a remarkable development for biofertilizer technology. Zhang et al. (2015) demonstrated that *B. amyloliquefaciens* (SQR9) is an ideal and more efficient PGPR than other strains of *Bacillus* strains (Table 2). Thirteen unique mobile genomic islands (GIs) were observed for the SQR9 strain. These GIs were found to be involved in the synthesis of many known and unidentified novel compounds. A recent report also demonstrated that maize root exudates regulate 98 genes in SQR9 for carbohydrate and amino acid metabolism. The set of the genome in SQR9 performed several functions like extracellular matrix production and regulated gene expression (Table 2), which revealed a high density (1.8 × 10^6^ CFU g^−1^ root) of SQR9 in the maize rhizosphere, and triggers plant growth (Zhang et al., 2015).

**Table 2:** Root exudates of maize plant recruit *B. amyloliquefaciens* strain. The whole genome sequence of *B. amyloliquefaciens* revealed that the group of genes induced by root exudates and its functional gene triggers rhizoadaptation, phytostimulation, bioferlilizer, and biocontrol activity.

**Gene**	**Functions**
*fZB42, ysnE, yhcX*, and *dhaS*	Tryptophane dependent indol-3-acetic acid (IAA)
*alsRSD* operon	2, 3-butanediol biosynthesis
*phy*	Phosphate solubilization
*gap* and *fbaB*	Embden-Meyerhof-Parnas pathway
*sucC*	Tricarboxylic acid cycle
*iol* cluster	Inositol
*mtlD*	Mannitol
*hxlA*	Hexulose
*dat, alaT*	Alanine
*gltD, gltA*	Glutamate
*kamA*	Lysine
*dapG*	Aspartate
*pTS*	Phosphotransferase system or sugar transporter
*citH*	Citrate transporter
*glnQHM, yveA, appC*, etc	Amino acid/Peptide
*yclN, yclO*	Ferrichrome
*sapB*	Mg^2+^
*pst* cluster	Phosphate
*epsA*-*O*	Exopolysaccharides synthesis
*tapAsipW*-*tasA*	Extracellular protein production
*bslA*	Self-assembling the bacterial hydrophobin that coats the biofilm
*spo0A* ~*p*-*AbrB*/*sinl*-*sinR*	Regulatory genes
*degQ*	Stimulates phosphotransfer from DegS ~ P to DegU; enhanced the biofilm formation and root colonization of SQR9
*bglC, bglS, bglA, licH* etc	Cellulose degradation
*xynA, xynB, xylR* and *xylAB*	Xylan transport
*bglS* and *bglA*	Significantly induced by root
*cheA, cheB, cheW, mcpB*, and *mcpC*	Involve in chemotaxis
*fliF*-*L, flgD, flgG, flhA, flhF*	Flagella synthesis

*(Source: Wang et al., 2009; Zhang et al., 2015; Wanga et al., 2018; Zhalnina et al., 2018)*.

## Mechanistic Insight of Plant Growth-Promoting Rhizobacteria

### Root Exudation

Narasimhan et al. (2003) reported two groups of root-exudates:(i) low molecular weight (LMW) such as sugars, amino acids, phenolics, secondary metabolites, and organic acids (citric, malic, oxalic, pyruvic, and succinic, etc.), and (ii) high molecular weight (HMW) that include proteins and complex carbohydrates. The nature and specificity of root exudates are dependent on the host species, plant developmental stages, physio-chemical nature of the soil, and surrounding microbial diversity (Hu et al., 2018; Singh et al., 2022). The maximum concentration of root exudates is found at the root tips and the lateral branching of the roots. Its amount also attenuates with increasing root surface (Compant et al., 2019), diffusion and degradation through sorption, deposition, or microbial consumption (Reinhold-Hurek et al., 2015). The microbial consumption contributes to the extravagance of root exudates owing to the valuable source of nutrition and energy for the rhizospheric microbes (Compant et al., 2019). The difference in the amount and nature of root exudates determines nutrient mobility, microbial population, and microbial diversity (Chamam et al., 2013; Bowya and Balachandar, 2020; Korenblum et al., 2020; Singh et al., 2022). Plant roots secrete root exudates in the rhizospheric region through passive (ion channels, vesicular transport, and diffusion) and active (secretion) mechanisms (Rohrbacher and St-Arnaud, 2016). LMW compounds are released through passive transport while HMW compounds through active transport mechanisms (Rohrbacher and St-Arnaud, 2016). The root exudates and solutes from cell membranes develop equilibrium between exterior and interior molecular transport (Weston et al., 2012; Cesari et al., 2019). In the passive mechanism, polar molecules and ions diffuse through the membrane using channels/permeases through a process called facilitated diffusion. These channels act as a passage for small ions like Na^+^, K^+^, Cl^−^, etc., and water, which aid in maintaining intra-cellular pH, membrane potential, osmotic status, and stabilized volume of the cell (Lee et al., 2007). The small polar and uncharged molecules can transport through direct passive diffusion depending on membrane permeability (Weston et al., 2012; Rohrbacher and St-Arnaud, 2016). The non-polar molecules pass through without using channels or transfer proteins (Weston et al., 2012). The electrochemical gradient arises owing to charged molecules or ions like amino acids, sugars, carboxylates ions, etc. (Rohrbacher and St-Arnaud, 2016). Passive transport across the membrane through channels is driven by an electrochemical gradient (Rohrbacher and St-Arnaud, 2016). Without any expense of energy, the movement based on an electrochemical gradient is called passive transport. The transport that requires energy from ATP for several ions or molecules against the concentration gradient or electrochemical gradient is called active transport (Rohrbacher and St-Arnaud, 2016). Plants have different coping mechanisms against the environment and secrete a large number of compounds that may require many transporters (Weston et al., 2012; Rohrbacher and St-Arnaud, 2016; Korenblum et al., 2020), and these transporters are capable of root exudation of aggregates into the rhizo-microbiome.

Weston et al. (2012) reported that the root exudates from root cells are transported by membrane transport proteins (MTPs). The ATP-binding cassette transporter helps in the phytochemical secretion from roots. Besides, Badri et al. (2013) have also described that out of 129 genes, 25 are significant for root exudation in *Arabidopsis thaliana*. A single gene mutation may influence the interaction among the microbial group of soil in *A. thaliana* (Badri et al., 2013). The MTPs include ABC transporter, multidrug and toxic compound extrusion (MATEs), major facilitator superfamily (MFS), and aluminum-activated malate transporter (ALMT). MATE transporter in rice root promotes exudation of polyphenolic compounds (Baetz and Martinoia, 2014). Recently, Wanga et al. (2018) reported that the aluminum exclusion from the root is facilitated by ALMT and citrate exudation through the MATE citrate transporter.

### The Action of Root Exudates

**Figure 2:**
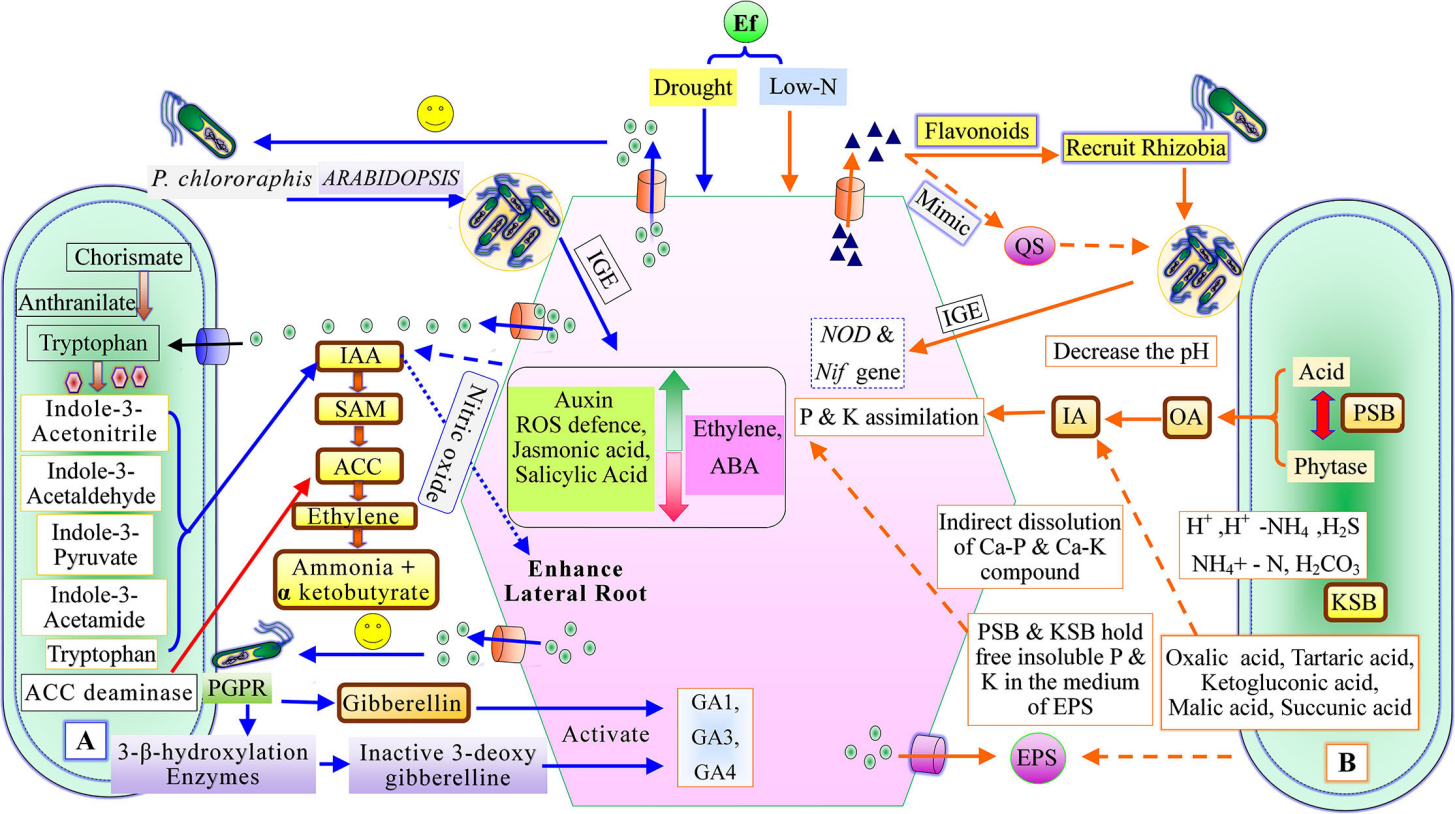
Plant growth-promoting mechanism of recruited plant growth-promoting rhizobacteria, **(A)** Phytostimulation, and **(B)** Biofertilizer activity [QS, Quorum sensing; IAA, indole-3-acetic acid; SAM, S-adenosyl methionine; ACC, 1-aminocyclopropane-1-carboxylate; IGE, Induce gene expression; ABA, Abscisic acid; GA1, GA2, and GA4, (Types of gibberellin); PSB, Phosphate solubilizing bacteria; KSB, Potassium solubilizing bacteria; EPS, exo-polysaccharides; OA, Organic acid; and IA, Inorganic acid].

Root exudates can mediate neutral, useful, or harmful interections between plant microbes and inter-species of microorganisms (Mendes et al., 2013; Hu et al., 2018). The secretion of root exudates rests on plant needs, and the rate of exudation is modified to cope with different biotic and abiotic stresses (Badri and Vivanco, 2009; Vardharajula et al., 2011). The root-driven changes in the microbial community observed by Donn et al. (2015) in the wheat rhizosphere demonstrated ten times more bacterial abundance than the bulk soil. Specific microbes like *Burkholderiales, Sphingobacterium*, and *Xanthomonadales* are dominant in the rhizospheres of *Brachypodium distachyon* in comparison to bulk soil (Kawasaki et al., 2016). Similar observations were recorded by Zhalnina et al. (2018) while studying the chemistry of root exudates of *Avenabarbata* where root-exudates were preferred as substrates for the specific bacterial community in the rhizospheres. The beneficial rhizobacterium *Pseudomonas putida* KT2440 is chemotactically attracted by 2-4-dihydroxy-7methoxy-1,4-benzoxazin-3-one from root exudates of *Zea mays* (Neal et al., 2012). The root exudate compounds like flavonoids act as signaling molecules, regulate *nod-*gene expression, activate *nod-*factors (lipochito-oligosaccharide), and trigger nodulation establishment in legumes (Abdel-Lateif et al., 2012; Figure 2). The flavonoids are released due to overcoming nitrogen deficiency in soil (Coronado et al., 1995). The flavonoid 7,4-dihydroxyflavone, from the root exudates of *Medicago sativa*, can mediate interaction with a diverse range of acid bacteria along with the induction of the *nod*-gene in the legumes (Szoboszlay et al., 2016). Strigolactone stimulates hyphal branching in mycorrhiza (Akiyama et al., 2005), and malic acid helps in the recruitment of plant growth-promoting rhizobacteria (Rudrappa et al., 2008). On the other hand, root-exudates have antimicrobial secondary metabolites such as benzoxazinoids (BXs), which can trim actinobacteria, proteobacteria, and pathogenic microbial populations in the maize rhizosphere (Hu et al., 2018). Root exudates influence the recruitment and make-up of microbiota in the plant rhizosphere (Hartmann et al., 2009; Ladyginaand Hedlund 2010; Reinhold-Hurek et al., 2015; Table 1). The ability of *A. brasilense* to modulate the plant root architecture was reported by Creus et al. (2005). Molina-Favero et al. (2008) observed that *A. brasilense* can synthesize nitric oxide (NO) aerobically, which mediates the IAA signaling pathway, leading to lateral and adventitious root formation in tomatoes. In *Arabidopsis*, under drought stress, root colonization of *P. chlororaphis* increases the expression of genes associated with ROS (reactive oxygen species) defense, auxin, jasmonic acid, and salicylic acid synthesis (Cho et al., 2013). *P. chlororaphis* also decreases the expression of ethylene and abscisic acid genes in *Arabidopsis* under drought stress (Cho et al., 2013; Figure 2). The roots of watermelon secrete more trans-chlorogenic acid and caffeic acid, followed by trans-cinnamic acid, which induced resistance against *Fusarium oxysporum* (Ling et al., 2013). Cai et al. (2009) reported that leguminous plant roots secrete canavanine, which recruits beneficial microorganisms. Canavanine favors the growth of selective rhizobia and also acts as an antimicrobial for pathogenic bacteria (Cai et al., 2009). Sugars and strigolactone, viz. 5-dexystrigal, components of non-legume root exudates mediate symbiotic association with mycorrhizal fungi (Fang and St Leger, 2010). Nguema-Ona et al., 2013 observed that AGPs (arabinogalactans protein) of root exudates also attract plant growth-promoting rhizobacteria through the chemo-attractant mechanism, and the maximum amount of AGPs was found at the root tip regions of the plants (Cannesan et al., 2012). AGPs induce a population of beneficial microbes in leguminous and non-leguminous plants (Xie et al., 2012; Vieira et al., 2020). The VOCs, *myc-*factors, *nod-*factors, exopolysaccharides, etc. are signaling components associated with rhizospheric microbes (Goh et al., 2013). VOCs (acetoin, 2-3-butanediol) mediate communication between plant microbes, induce ISR (induced systemic resistance) as bio-protestants (Ryu et al., 2004), and plant growth promotion. Ankati and Podile (2019) reported that threonine and glyoxylic oxime acid from root-exudates of groundnut influenced *Pseudomonas aeruginosa* (RP2), while serine, pentanoic acid, glucopyranoside, tartaric acid, and 2-pyrrolidinone influenced both *P. aeruginosa* (RP2) and *B. sonorensis* (RS4). These findings demonstrated that a specific component of root-exudates was responsible for selective PGPR interaction. Thus, the products of root exudates could be an effective agent for improving crop yield at the field level by enhancing PGPR colonization.

Quorum sensing (QS) helps in establishing root microbe assemblage in the rhizosphere. The root exudates mimic QS signals of bacteria to repress QS-regulated responses of associated/adjacent bacteria. The root exudates have primary and secondary metabolites along with proteins (Korenblum et al., 2020). Some reports demonstrated that these proteins influenced the selective recruitment of useful bacteria (De-la-Pena et al., 2008). The QS compound in the root exudates of groundnut plants selects microbes and induces their population (Ankati and Podile, 2019). Bacteria communicate within the system through a density-dependent mechanism known as QS (Reinhold-Hurek et al., 2015). The QS regulates the metabolic as well as the behavioral activities of the bacterial community (Marketon et al., 2002; Nievas et al., 2012; Liu et al., 2014; Korenblum et al., 2020). This sort of interaction occurs through a dialect of chemical signals called autoinducers (AHLs: acyl homoserine lactones), autoinducing peptides (AIP), and autoinducer-2; furanone (AI-2), synthesized by bacteria (Figure 1). AHLs mediate signaling in gram-negative bacteria (Vial et al., 2006). AIP requires specialized membrane transport protein for signaling in the gram-positive bacteria, whereas AI-2 is required for both gram-positive and gram-negative bacteria (Abisado et al., 2018). Bacterial QS occurs through various complex pathways depending upon species diversity (Reinhold-Hurek et al., 2015). Therefore, the cognizance of the QS enables the regulation, thereby constraining bacterial communication (Figure 1). The inhibition strategies of QS are jointly called quorum-quenching, through which bacteria are ineffective in their interplay with each other. QS-mediates bacterial processes like growth, conjugation, bioluminescence, biofilm formation, siderophore production, and swarming (Barriuso et al., 2008). The threshold level of the initial plant growth-promoting rhizobacteria inoculum mediated by QS molecules strongly induces plant growth performance (Rodriguez et al., 2020). The plant rhizospheric region has a higher amount of AHL in comparison to the bulk soil, suggesting that these trigger bacterial colonization and establish a strong association between bacteria and plant roots (Vial et al., 2006).

### Plant Growth-Promoting Rhizobacteria

**Figure 3:**
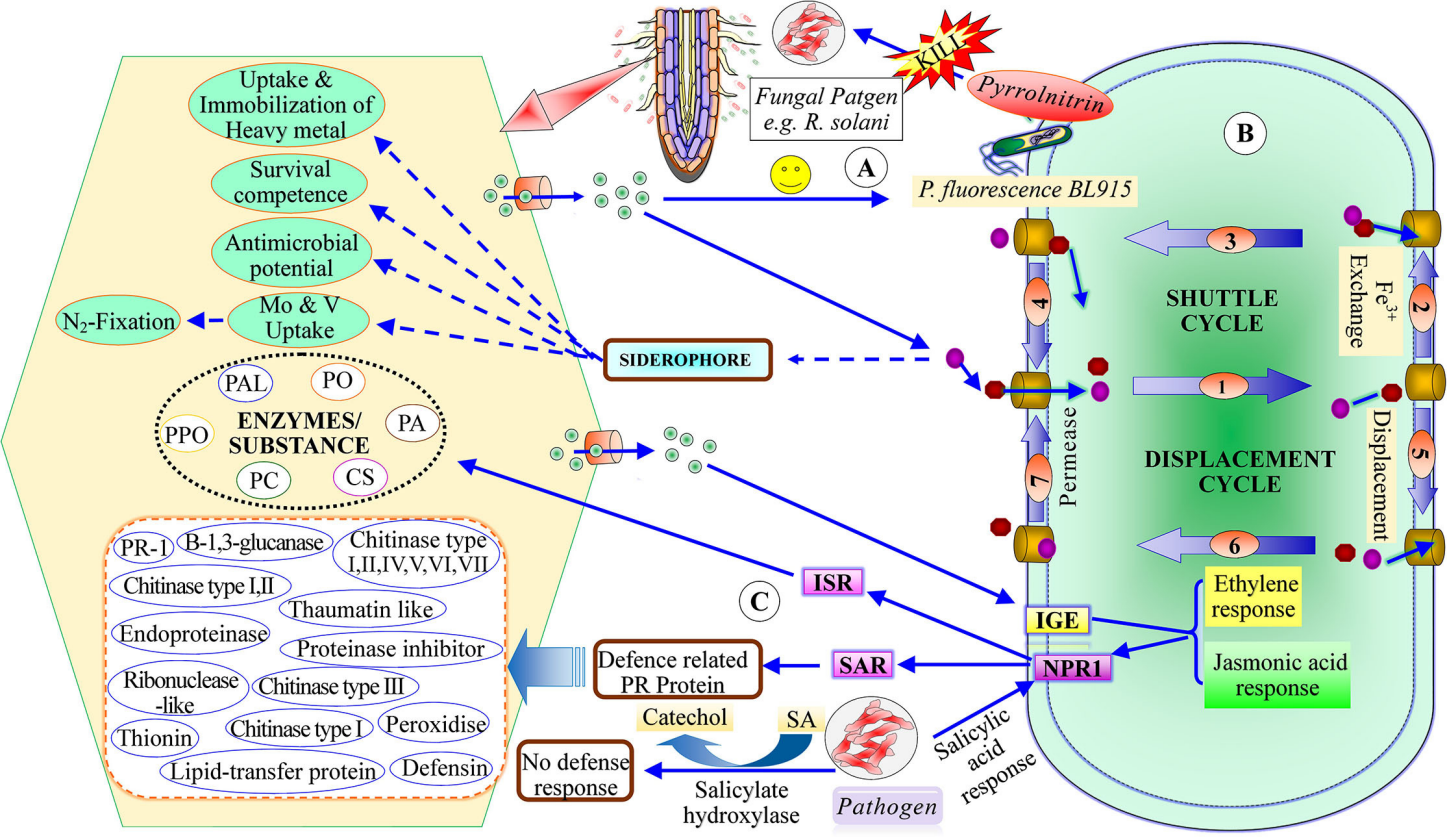
Indirect mechanism of plant growth-promoting rhizobacteria **(A)** recruited PGPR to produce antibiotic **(B)** siderophore production and plant growth-promotion, **(C)** mechanism of induced systematic resistance [ISR, induced systemic resistance; SAR, Systematic acquired resistance; IGE, Induce gene expression; NPRI, Non-expresser pathogenesis-related gene; SA, salicylic acid; PAL, phenylalanine amonislyase; PO, peroxidase; PPO, polyphenol oxidase; PC, poly-phenolic compounds; CS, chalcone synthase; PA, phytoalaxine.

Menendez and Garcia-Fraile (2017) classified plant growth-promoting rhizobacteria into extracellular plant growth-promoting rhizobacteria (e-PGPR) and intracellular plant growth-promoting rhizobacteria (i-PGPR). The plant growth-promoting rhizobacteria stimulate plant growth directly by the activity of phytostimulation and bio-fertilization, whereas indirectly through biopesticides or bio-control agents (Dwivedi and Dwivedi, 2002; Glick et al., 2007; Glick, 2012; Ngoma et al., 2012). The direct mechanism of plant growth-promoting rhizobacteria facilitates nutrient uptake or improvement in nutrient availability by nitrogen fixation (Cheng, 2008; Glick, 2012), solubilization of phosphorus and mineral nutrients, mineralizing organic compounds (Khan et al., 2010; Sharma et al., 2013), and phytohormones production including, IAA, ethylene, cytokinins, and gibberellins (Pliego et al., 2011; Upadhyay et al., 2016, 2019; South et al., 2021; Figure 2). On the basis of PGPR function, siderophore production may be considered as both a direct and an indirect mechanism (Ahmed and Holmstrom, 2014). The indirect mechanisms include antibiotic production (Sindhu et al., 2009), hydrolytic enzyme production (Dubey et al., 2014), induced systemic resistance (ISR), and exo-polysaccharides (EPS) production (Upadhyay et al., 2011; Figure 3).

## Direct Mechanisms

### IAA Production

IAA (indole-3-acetic acid), a product of the amino acid L-tryptophan (Yu et al., 2017), acts as a plant growth regulator. The IAA regulates the plant-growth through several cellular functions such as cell division, cell elongation and differentiation, increase in root length and root surface area, and the development of flowers (Gravel et al., 2007; Santner et al., 2009). About 80% of rhizobacteria produce auxins of microbial origin but with analogous functions, such as the auxins of plant origin (Patten and Glick, 1996; Ahemad and Kibret, 2014; Myresiotis et al., 2015; Keswani et al., 2020b. The IAA plays a crucial role in the interaction between plant and rhizobacteria and is synthesized by the associated plants and many microbes like plant growth-promoting rhizobacteria (Upadhyay et al., 2009). Several studies have demonstrated that the IAA production system was present in many bacterial species such as *Agrobacterium tumefaciens, Alcaligenes faecalis, Agrobacterium tumefaciens* (Costacurta and Vanderleyden, 1995), *Pseudomonas syringae* (Kosuge and Sanger, 1987), *Streptomyces* sp., *B. subtilis* spp. (Swain et al., 2007), *Pseudomonas fluorescens* (Oberhansli et al., 1991), and *B. megaterium* (Nghia et al., 2017).

### Ethylene Production

Ethylene is a unique type of plant hormone secreted by plants and plant growth-promoting rhizobacteria (Babalola, 2010). Ethylene regulates plant physiological processes such as seed dormancy, enhances the formation of an adventitious root, leaf abscission, senescence of flower and leaf, and fruit ripening (Abeles et al., 1992). Different environmental conditions like salinity, drought, low temperature, pathogenic attack, and chemical exposure alter ethylene production and plant growth (Babalola, 2010). The optimum concentration of ethylene induces positive growth and development of plants (Saleem et al., 2007). Ethylene production directly depends on ACC production (Shaharoona et al., 2006).

### Gibberellins and Cytokinin Production

Gibberellin (GA) is a tetracyclic di-terpenoid compound that acts as a plant hormone and is synthesized by many plant growth-promoting rhizobacteria. GA regulates many plant functions, such as fruit ripening, cell division, plant growth, etc. (Martinez et al., 2016; Plackett and Wilson, 2016). The endogenous GA concentration is raised by GA-producing plant growth-promoting rhizobacteria in the rhizospheric zone and it induces plant growth; for example, *Leifsonia soli*-SE134 and *Enterococcus faecium*-LKE12 in rice plant (Kang et al., 2014; Lee et al., 2015). Cytokinin is a plant hormone and is also synthesized by plant growth-promoting rhizobacteria. It is a member of adenine derivatives of the N-6 substituted group (Tsukanova et al., 2017), promotes cell cycle of the plant (Schaller et al., 2014), regulates plant growth and biosynthesis of chlorophyll (Cortleven and Schmülling, 2015). Plant growth-promoting rhizobacteria can produce Cytokinin and cause an increase in plant growth even under stressed conditions (O'Brien and Benkova, 2013). Tahir et al. (2017) demonstrated that *B. subtilis*-SYST2 induces the expression of cytokinin gene (*s1CKX1*) in tomato plants (Tahir et al., 2017). The plant growth-promoting rhizobacteria like *Rhizobium* sp., *Azotobacter* sp., *Pantoea agglomerans, Pseudomonas fluorescens, Rhodospirillum rubrum, B. subtilis*, and *Paenibacillus polymyxa* secrete cytokinins and/or gibberellins that promote plant growth (Kang et al., 2010).

### Availability of Nutrients

The soil acts as a buzzword source of macro- and micro-nutrients for plant growth, while the compatible form of nutrients is a question of their availability to the plant. Most of the microorganisms can enhance the availability of nutrients in their compatible form to the plants (Upadhyay et al., 2019). Thus, soil fertility is one of the significant factors that governs diverse mechanisms of microorganisms. Nitrogen occurs in 78% of total atmospheric gases and cannot be utilized by higher plants directly. The nitrogen is utilized when it becomes fixed in the form of nitrogenous salt or ammonium ion. In nature, two kinds of N_2_ fixation occurs i.e., biological and non-biological (Raza et al., 2021). The biological nitrogen fixation (BNF) is carried out with the aid of bacteria, fungi, and algae, etc., which makes the atmospheric nitrogen available in the form of nitrogenous salt through the action of several plant growth-promoting rhizobacteria and blue-green algae (Dwivedi and Dwivedi, 2004). The BNF is borne out by two kinds of microbes, e.g., symbiotic and non-symbiotic (Tang et al., 2020; Raza et al., 2021). Symbiotic-BNF, a mutualistic link between the microbe and the plant, occurs in leguminous plants such as pea, chickpea, etc. (Cheng, 2008). In symbiotic-BNF, microbes enter the root and induce nodule formation (Cheng, 2008; Singh et al., 2022). Free nitrogen peroxide is converted into ammonia by nitrogen-fixing microorganisms and makes it available to the host (Ahemad and Kibret, 2014). This process involves a complex enzyme system known as nitrogenase (Gaby and Buckley, 2012), and the *nif*-gene, found in symbiotic as well as free-living bacterial systems (Reed et al., 2011). Nitrogen-fixing plant growth-promoting rhizobacteria such as *Azospirillum* (Montanez et al., 2009), *Klebsiella* (Arruda et al., 2013), *Burkholderia* (Chelius and Triplett, 2001), *Bacillus* (Zakry et al., 2012), and *Pseudomonas* (Piromyou et al., 2011) can significantly enhance crop productivity. *Bacillus* is associated with N_2_-fixing bacteria promoting plant growth and enhances the yield in non-leguminous cereals (Cakmakci et al., 2001; Ramirez and Mellado, 2005) such as maize (Pal, 1998), sugar beet, and barley (Sahin et al., 2004).

Mostly, phosphorus exists in the insoluble complex form like calcium phosphates in saline soil (Goldstein and Krishnaraj, 2007) and iron phosphates and aluminum phosphates in acidic soil (Mullen, 2005). Phosphorus is commonly present in soil in the range of 400–1,200 mg kg^−1^ (Fernández et al., 1988). Earth rocks are rich sources of phosphorus, in the form of primary apatites, and other primary minerals that previously existed in the geological age (Fernández et al., 1988). Indian soils are commonly deficient in phosphorus (Johri et al., 2003), and there are about 40 million tons of phosphorus deposits in India (Roychoudhury and Kaushik, 1989). Phosphate solubilizing bacteria (PSB), actinomycetes, and phosphate solubilizing fungi (PSF) solubilize the complex form of phosphates in the soil (Khan et al., 2007). The plant growth-promoting rhizobacteria induce the availability of phosphorus through mineralization and solubilization of the compounds of rock phosphates (organic and inorganic phosphorus; Nahas, 1996; Hilda and Fraga, 2000; Khiari and Parent, 2005). Bacterial species are superior phosphorus solubilizers than fungal species (Alam et al., 2002); PSB involves about 1–50% phosphorus solubilization followed by PSF, i.e., about 0.1–0.5% (Chen et al., 2006). *Pseudomonas, Bacillus, Rhizobium*, and *Enterobacter*, and fungal genera such as *Penicillium* and *Aspergillus* are the most efficient phosphate solubilizers (Kucey et al., 1989; Rodriguez and Fragal, 1990).

About 90% of potassium (K) in the soil exists in its complex form and is not available to the plants (Yadegari and Mosadeghzad, 2012; Zhang et al., 2013). Therefore, the solubilization of potassium is essential for K uptake by plants. K enhances seed germination, plant growth productivity, seedling vigor, and plantbiomass (Awasthi et al., 2011; Lynn et al., 2013; Meena et al., 2014; Zhang and Kong, 2014). The plant growth-promoting rhizobacteria can solubilize potassium rock (e.g., biotite, feldspar, illite, muscovite, orthoclase, and mica) into an available form of potassium for the plant. Potassium solubilizing plant growth-promoting rhizobacteria releases organic acids (e.g., oxalic acid, tartaric acids, gluconic acid, 2-ketogluconic acid, citric acid, malic acid, succinic acid, lactic acid, propionic acid, glycolic acid, malonic acid, fumaric acid, etc.) and inorganic acids (Awasthi et al., 2011; Etesami et al., 2017), which play an effective role in releasing K from K-bearing minerals (Hu et al., 2006; Liu et al., 2012; Keshavarz et al., 2013; Saiyad et al., 2015).

Different types of organic acids are involved in potassium solubilization, but the most prominent acids are tartaric acid, citric acid, succinic acid, α-ketogluconic acid, and oxalic acid released by KSB (Meena et al., 2014). Both aerobic and anaerobic plant growth-promoting rhizobacteria act as KSB, but most frequently aerobic bacteria that act as potassium solubilizers are *Acidothiobacillus ferrooxidans, B. edaphicus, B. mucilaginosus, Burkholderia, Paenibacillus* sp., and *Pseudomonas* (Etesami et al., 2017). Saprophytic bacteria, fungal strains, and actinomycetes also participate in K solubilization in a wide range (Gundala et al., 2013; Meena et al., 2014; Bakhshandeh et al., 2017). Thus, KSB–PGPR acts as a biofertilizer that improves soil fertility and plant growth. KSB is commonly found in different soil environments and can be isolated from rhizospheric and non-rhizospheric soil, including paddy soil (Bakhshandeh et al., 2017) and saline soil (Bhattacharya et al., 2016). KSB–PGPR is more effective for potassium solubilization (4.90 mg l^−1^) at a specific pH range of 6.5–8.0 (Badr et al., 2006). Similarly, *Bacillus* sp., *Burkholderia* sp., and *Pseudomonas* sp. can solubilize potassium at different temperatures and carbon sources from tea (*Camellia sinensis;* Bagyalakshmi et al., 2012).

## Direct/Indirect Mechanism

### Siderophore Production

Plant growth-promoting rhizobacteria secrete a low molecular weight (500–2,000 Da) iron-chelating compound called siderophore (Ahmed and Holmstrom, 2014). Siderophores are involved in the transportation and uptake of iron elements in the plant cells (Singh et al., 2022) and induce plant growth (Schwyn and Neilands, 1987; Hider and Kong, 2010; Ahmed and Holmstrom, 2014). *Pseudomonas* sp., *Enterobacter* genera, *Bacillus*, and *Rhodococcus* have a special capacity for binding iron through siderophores or siderochromes (Sah and Singh, 2015). Those microorganisms which cannot produce siderophores but use siderophores produced by other microorganisms are called xenosiderophores (Ahmed and Holmstrom, 2014). Production of siderophores occurs at specific conditions such as pH, temperature, and iron-concentration. The bacteria *P.chlororaphis* PCL1391 strain of rhizospheric roots of tomato plants can solubilize iron from the insoluble ferric oxides at neutral pH (Hernandez et al., 2004; Haas and Defago, 2005). Similarly, Sinha et al. (2018) reported that *Psychrobacter piscatorii* and *Enterococcus casseliflavus* from the Kerguelen Islands and *B. cereus, Pseudoalteromonas tetraodonis, Psychrobacter pocilloporae, Pseudomonas weihenstephanensis*, and *Micrococcus aloeverae* from the Prydz-Bay produced either hydroxamate-type siderophore or catecholate-type siderophores at 15–25°C with pH 8.5. Siderophores are produced by both aerobic and facultative anaerobic types of bacteria under the iron stress habitats (Neilands, 1995). Facultative aerobic bacterium such as *Pseudomonas stutzeri* CCUG 36651 produced siderophores under both aerobic and anaerobic conditions (Essen et al., 2007), *Pseudomonas stutzeri* CCUG 36651 produced four types of ferrioxamine siderophores under aerobic conditions but ferrioxamines siderophores under anaerobic conditions (Essen et al., 2007). Siderophore tightly binds with iron (Fe^+3^), then Fe^+3^-chelates move inside the cell through the cell membrane with the help of specific siderophore receptors (Haas and Defago, 2005). There are several types of siderophore binding proteins, such as permeases and ATPases involved in the transport of Fe^+3^ chelating compounds in the cell membrane, reported in gram-positive bacteria (Ahmed and Holmstrom, 2014). Gram-negative bacteria release many enzymes including periplasmic binding protein, outer membrane receptors, and cytoplasmic membrane protein, which help in the transport of iron-chelating compounds (Ahmed and Holmstrom, 2014).

### Indirect Mechanisms of Plant Growth-Promoting Rhizobacteria

Indirect mechanisms involve antibiotics production, and hydrogen cyanide (HCN), ISR, and EPS production. The secretion of lytic enzymes of plant growth-promoting rhizobacteria induces plant growth (Maksimov et al., 2011; Upadhyay et al., 2016). The production of antibiotics is one of the most powerful bio-control tools for plant pathogens. Antibiotics are heterogeneous, low molecular weight, organic compounds secreted by microorganisms that help plant growth and metabolic activities (Duffy, 2003). The first antibiotic used as bio-control for plants was isolated from the bacterial species of fluorescent pseudomonads (Weller and Cook, 1983). Based on the mode of action, there are six classes of antibiotics, namely, phenazines, phloroglucinols, pyoluteorin, pyrrolnitrin, cyclic lipopeptides, and hydrogen cyanide (Haas and Defago, 2005). A large number of bacterial and fungal species secrete various types of antibiotics which induce plant growth by the suppression of phytopathogens (Maksimov et al., 2011). Pyrrolnitrin, an antibiotic isolated from *P. fluorescens* BL915 strain suppresses the growth of the fungal pathogen *Rhizoctonia solani* in cotton plants (Hill et al., 1994). Bacterial species of *Pseudomonas* secretes phenazine antibiotics that suppress various fungal pathogens including *F. oxysporum* and *Gaeumannomyces graminis* (Chin-A-Woeng et al., 2003). *Bacillus* sp. produces many types of antibiotics such as polymyxin, circulin, and colistin that suppress many plant diseases (Maksimov et al., 2011).

Hydrogen cyanide is a volatile secondary metabolite secreted by several gram-positive and gram-negative bacteria such as *P.fluorescens, P. aeruginosa, Chromobacteria violaceum*, etc. (Morrison and Askeland, 1983) that act as bio-control agents against soil-borne phytopathogens (Haas and Defago, 2005). HCN acts as a powerful inhibitor of various metallic enzymes including copper-bearing cytochrome C oxidase (Cho et al., 2013). HCN prevents many plant diseases like root-rot and black-rot diseases of tomato plants (Voisard et al., 1989) and also has nematicidal activities (Kang et al., 2010; Anderson and Kim, 2018). It is also very useful in agriculture and forestry due to the control of subterranean termites, *Odontotermes obesus* (Devi et al., 2007).

Induced systemic response (ISR) suppresses the disease of plants and animals that induces resistance against diseases (Van Loon et al., 1998). ISR induced by rhizobacteria shows resistance to several pathogens such as bacteria, fungi, and viruses (Korenblum et al., 2020). The plant growth-promoting rhizobacteria strain secretes salicylic acid that produces resistance to plant diseases, indicating that PGPR induces ISR (Krause et al., 2003; Idris et al., 2004). The treatment of tobacco plants with *Bacillus* rhizobacteria suppressed the impact of TMV (Tobacco Mosaic Virus) and also enhanced the height, weight, and yield of tobacco plants (Kloepper et al., 2004; Wang et al., 2009).

Exo-polysaccharides is a very active constituent of soil organic matter (Gouzou et al., 1993) produced by plant growth-promoting rhizobacteria under different soil environments like salinity (Upadhyay et al., 2011; Mohammed, 2018), drought, and normal conditions (Alami et al., 2000). EPS is the most important component of the extracellular matrix that shows two characters, slimy EPS and capsular EPS. EPS plays a significant role in several functions like biofilm formation (Bhaskar and Bhosle, 2005), bacterial cell protection (Mohammed, 2018), pollutants degradation (Fusconi and Godinho, 2002), plasma substituting capacity and bioremediation (Mohammed, 2018), maintenance of primary function of the cell, and antibacterial activity (Alami et al., 2000; Mohammed, 2018). EPS-producing PGPR influences root-adhering soils and establishes a balance between plant roots and microbial populations (Alami et al., 2000; Upadhyay et al., 2011).

Hydrolytic enzymes (HEs), mainly chitinase, glucanase, protease, and cellulase can hydrolyze chemical bonds of a wide variety of polymeric compounds including chitin, proteins, cellulose, hemicelluloses, and phytopathogenic DNA (Jadhav and Sayyed, 2016). HEs are capable of controlling phytopathogens through the hydrolysis of the cell wall, proteins, and DNA of pathogens. Thus, HEs play a major role in bio-control (Prathap and Ranjitha, 2015; Jadhav and Sayyed, 2016). The plant growth-promoting rhizobacteria act as an effective BCA through the lysis of phytopathogenic DNA (Garbeva et al., 2004). Microbial strains such as *S. marcescens, B. cereus, B. subtilius*, and *B. thuringiensis* can produce HEs that can control several phytopathogens namely *R. solani, F. oxysporum, S. rolfsii, P. ultimum*, etc. through different mechanisms (Someya et al., 2000).

### Selective Recruitment of Plant Growth-Promoting Rhizobacteria

It is appealing to discuss what triggers the recruitment of microbiome in the rhizosphere. Microbiome in the rhizosphere affects plant growth and yield of the crop. There are two possibilities: (i) plant root creates an environment in the rhizosphere and attracts the useful microorganism/bacteria in the rhizosphere, (ii) soil already has a specified microbial population that allows the growth of selective plants. Here, we will discuss the first possibility which is more relevant to the root exudates and relevant to the remit of this review. Several previous reports demonstrated that the composition of root exudates varies with plant species, soil type, pH, and developmental stage (Berg and Smalla, 2009). The specific components of plant exudates promote the recruitment of specific microbiome/PGPR (Kamilova et al., 2006a). It is well-recognized that the microbial community in the rhizosphere is highly distinguished in different plant species (Edwards et al., 2015), the reason being the availability of different genotypes of the host plant. Based on the knowledge till date, the repertoire of the microbial community (especially the PGPR) around the plant root can be managed. It has been stated that some workers induced the recruitment of plant growth-promoting rhizobacteria by mutating the ABC transporter gene in plants (Badri et al., 2009). These results suggest the growth of a disease-resistant plant by influencing the root exudate components for the recruitment of plant growth-promoting rhizobacteria (Wei and Jousset, 2017). Natural disease-suppressive soil (term defined by Baker and Cook, 1974) can be achieved by manipulating the plant exudate resulting in the recruitment of the required PGPR (Exposito et al., 2017). Thus, differential components in plant exudates recruit microbial communities with a certain degree of specificity.

## Future Perspectives

Many pieces of research demonstrated the diverse compounds of root exudates and their sensing toward beneficial microbes studied under confined and controlled laboratory conditions. Therefore, elucidation of the function of chemotaxis behavior of microbe-mediated compounds of root exudates is necessary for future research to make the success story at the field level. This will provide the structural foundation for a wide range of PGPR recognition by specific compounds (chemoattractants) of plant root exudates, respectively, and induce the growth of sustainable agriculture by chemotaxis to genetically modified plant growth-promoting rhizobacteria under degraded soil. Despite little knowledge of chemoattractant compounds of plant's root exudates, there are scopes for more researches for getting diverse advantages of root exudates through the application of emerging technology. Biotechnology is the utilization of biological resources for human welfare and industrial use. Plants have a well-developed system for the secretion of root exudates. Whether this secretion system can be utilized for biotechnological application is the central issue. Undoubtedly, there are several published reports, but the most fitting domains are (i) rhizosecretion and (ii) phytoremediation. Rhizosecretion is an alternative platform for manufacturing a large amount of pure target proteins (Drake et al., 2009). Borisjuk et al. (1999) demonstrated the production of recombinant proteins in plant root exudates. For this purpose, a genetically engineered plant with increased root mass can be used (Gaume et al., 2003). Rhizosecretion has been utilized for the production of antibodies. Madeira et al. (2016) have demonstrated that hyposecretion is an efficient and economical method for monoclonal antibody production. Catellani et al. (2020) recently evaluated the production of anti-fungal antibody scFvFc 2G8 using the root hair secretion system in *Nicotiana benthamiana* and *Solanum lycopersicum*. Another example of next-generation human therapeutic antibody production was demonstrated by Lonoce et al. (2016). They showed the production of tumor-targeting human-compatible monoclonalantibody H10 in hairy root plants (Lonoce et al., 2016).

Environmental pollution is the most devastating condition in the present ecological perspective. Phytoremediation is the process of removal of water and soil contaminants, especially by using the plant root system (Upadhyay and Edrisi, 2021). The role of root exudate in the removal of soil and water contaminants has been reported outstandingly (Gleba et al., 1999; Ma et al., 2016; Chen et al., 2020). Different components of root exudates play a specific role in the removal of certain contaminants from the soil and groundwater. Lu et al. (2017) reported that glucose present in plant exudates can remove the soil pyrene by promoting soil dehydrogenase activity. Palmitic acid present in plant exudates of tall fescue showed promising results in the removal of petroleum contaminants from the soil (Liu et al., 2015). Similarly, by manipulating the components of root exudates, it can be used for the removal of targeted pollutants from the soil and ground water. There are two main types of phytoremediation processes exploiting the plant exudates (i) rhizosphere biodegradation (plant root exudate recruited microbe mediated degradation of pollutants) and (ii) phytostabilization (plant exudate components immobilize the pollutants).

## Concluding Remarks

Since the 1980s, plant growth-promoting rhizobacterial inoculants have been developed, but few of them revealed irregular performance at the field level. Although several researchers have developed the consortia of plant growth-promoting rhizobacteria, but with more or less similar outcomes in the farmer's field, the solution to these problems is somehow hidden in the root exudates and root microenvironment. Thus, the present review has concentrated on the remarkable views for future research to manage the challenges at the field level with PGPR inoculants. Several components of root exudates have functional interplay with PGPR either directly or through their gene expression. The recruitment of plant growth-promoting rhizobacteria through root exudates can enhance plant growth-promoting rhizobacteria root colonization, specifically, and induce close sustainable relationships between them for a long time. The hypothesis of specific recruitment would address the key gap for warranting the perfect plant growth-promoting rhizobacteria candidate and opening a new horizon of research in biofertilizer technology. It would be a promising technique for reducing the asymmetrical performance of plant growth-promoting rhizobacteria in the farmer's field.

## Author Contributions

SU, AS, PC, PD, and AB contributed to conceptualization and visualization of the present review and writing the original draft. SU and AS designed the figures and tables. DJ, PD, and GC contributed to re-structuring the review. PD, BS, VDR, and TM contributed special remarks and edited the review for final submission. All authors contributed to the article and approved the submitted version.

## Conflict of Interest

The authors declare that the research was conducted in the absence of any commercial or financial relationships that could be construed as a potential conflict of interest.

## Publisher's Note

All claims expressed in this article are solely those of the authors and do not necessarily represent those of their affiliated organizations, or those of the publisher, the editors and the reviewers. Any product that may be evaluated in this article, or claim that may be made by its manufacturer, is not guaranteed or endorsed by the publisher.
